# Audit of Blood Component Utilization and Request Appropriateness in a Tertiary Care Hospital: A Cross-Sectional Study

**DOI:** 10.7759/cureus.115020

**Published:** 2026-08-23

**Authors:** Vidhi Jain, Devanshi Gosai, Dipali Shukla

**Affiliations:** 1 Transfusion Medicine, Sumandeep Vidyapeeth Deemed to be University, Vadodara, IND; 2 Pathology, Sumandeep Vidyapeeth Deemed to be University, Vadodara, IND

**Keywords:** blood component audit, crossmatch-to-transfusion ratio, fresh frozen plasma, hemovigilance, hospital transfusion committee, india, packed red blood cells, platelet utilization, request appropriateness

## Abstract

Background: Blood components, packed red blood cells (PRBCs), fresh frozen plasma (FFP), and platelet concentrates (PCs), are finite resources whose inappropriate use compromises patient safety and depletes inventory. Structured audit of transfusion practice underpins sound hemovigilance, yet its application across Indian tertiary centers remains inconsistent. This study quantified the appropriateness of blood component requests and mapped component-wise and departmental variation.

Methods: A six-month retrospective cross-sectional audit (July-December 2025) was conducted at a tertiary care hospital blood bank. All blood component requests (n=8,752) were reviewed by two postgraduate pathology trainees, with senior specialist adjudication of disagreements, against prespecified operational criteria adapted from National Blood Transfusion Council (NBTC) India, British Committee for Standards in Hematology (BCSH), and American Association of Blood Banks (AABB) guidance. The crossmatch-to-transfusion (C:T) ratio was calculated for PRBCs, and request appropriateness was compared across departments using the chi-squared test, with Fisher's exact test where expected cell counts were small and Benjamini-Hochberg correction for multiplicity; proportions are reported with 95% confidence intervals (CIs).

Results: Of 8,752 requests, 6,202 (70.9%; 95% CI: 69.9-71.8) met appropriateness criteria, and 29.1% did not. FFP requests showed the lowest appropriateness (35.2% not meeting criteria), followed by PRBC (28.7%) and PC (21.6%). The overall PRBC C:T ratio was 2.1:1. Request appropriateness varied significantly across departments (χ²=161.3; df=6; p=3.2×10⁻³²), with Oncology (82.2%) and ICU/Critical Care (81.1%) showing the highest crude rates and Obstetrics and Gynecology (64.1%) and the heterogeneous "Others" group (63.3%) the lowest; these comparisons are unadjusted for component mix and within-patient clustering. Hemoglobin above threshold, FFP requested for volume support without documented coagulopathy, and missing clinical justification were the leading documented reasons among requests not meeting criteria.

Conclusion: A substantial proportion of blood component requests, particularly for FFP, did not meet prespecified appropriateness criteria, and appropriateness varied markedly across departments and components. These findings support the consideration of a structured, recurring institutional audit-and-feedback program; the effect of such a program was not tested in this study.

## Introduction

Blood transfusion sits close to the line between benefit and harm: given without a sound clinical reason, it exposes patients to risk rather than benefit [1]. Patient blood management, the coordinated set of practices intended to ensure that blood components are used only when clinically indicated, at the lowest effective dose, and with alternatives considered first, has been promoted internationally as a framework for reducing this risk while protecting a finite and sometimes scarce resource. Inappropriate component ordering carries consequences beyond the individual patient: it exposes patients to needless risks of transfusion reactions and alloimmunization, depletes inventory that may be needed more urgently elsewhere, and adds avoidable cost to already stretched blood banking services. Yet auditing whether component requests meet accepted criteria remains inconsistent across Indian tertiary centers, and the available evidence is largely retrospective, confined to a single component or department, and modest in size [2,3]. Reported appropriateness in existing Indian audits spans a wide range, and few studies test how appropriateness varies between departments within a single institution or quantify the specific, documented reasons why requests fail to meet criteria. This leaves a gap: institutions considering where to target audit-and-feedback efforts have little hospital-wide, component-by-component evidence to draw on. We therefore audited all three major blood components across every clinical department in a large teaching hospital, at scale, to locate and size the drivers of requests not meeting appropriateness criteria.

The primary objective of this audit was to determine the appropriateness of blood component requests (packed red blood cell (PRBC), fresh frozen plasma (FFP), and platelets) against prespecified criteria in a tertiary care hospital. The secondary objectives were to (i) assess departmental variation in request appropriateness, (ii) identify and quantify the documented reasons for requests not meeting appropriateness criteria, and (iii) evaluate the PRBC crossmatch-to-transfusion (C:T) ratio.

## Materials and methods

Study design and setting

This retrospective, cross-sectional audit covered six consecutive months, from July through December 2025, within the Department of Transfusion Medicine and Blood Centre of Dhiraj Hospital, a tertiary care teaching hospital located in Piparia, Vadodara, Gujarat, India, with a licensed strength exceeding 1000 beds.The institution runs full-fledged medicine, surgery, obstetrics, oncology, pediatric, and critical care services, generating a steady and substantial demand for blood components.

Participants

**Figure 1:**
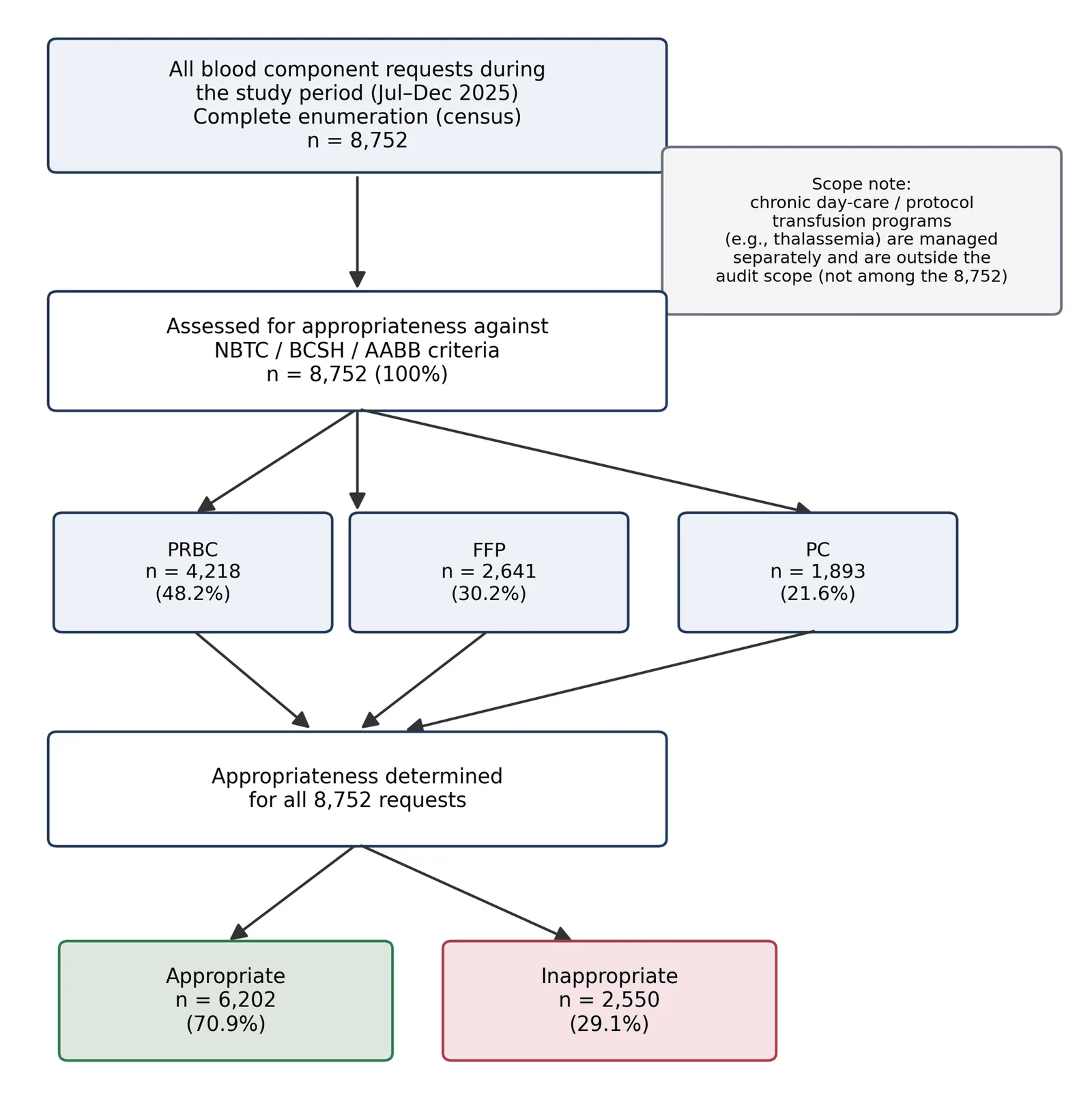
STROBE participant flow diagram All blood component requests during the study period were analyzed by complete enumeration (census; n=8,752); chronic day-care/protocol transfusion programs (e.g., thalassemia) are managed separately and fall outside the audit scope. Requests were assessed for appropriateness and broken down by component (PRBC n=4,218; FFP n=2,641; PC n=1,893) and by outcome (appropriate n=6,202 (70.9%); inappropriate n=2,550 (29.1%)). PRBC: packed red blood cell; FFP: fresh frozen plasma; PC: platelet concentrate; STROBE: Strengthening the Reporting of Observational Studies in Epidemiology; NBTC: National Blood Transfusion Council; BCSH: British Committee for Standards in Hematology; AABB: American Association of Blood Banks

Every inpatient and emergency request for a blood component logged during the study window was eligible for inclusion. Patients on chronic, pre-protocolized transfusion programs managed in day-care settings (such as thalassemia recipients) were left out of the appropriateness analysis, since their transfusion decisions are governed separately by the hematology/oncology unit rather than by the general criteria applied here. No sampling was undertaken: every eligible request in the study window was included, giving a complete enumeration (census) of 8,752 requests carried forward for analysis; participant flow is summarized in Figure 1.

Data collection

Data were extracted retrospectively from four existing record sources: archived requisition slips, the crossmatch register, the blood bank information system (BBIS), and ward-level clinical records. The unit of analysis was the individual component request, a single requisition for one component type for one patient at a given time, corresponding to one transfusion episode rather than a unit-level or patient-level event. A patient could contribute more than one request over the study period; requests were treated as independent events and patient-level clustering was not statistically modelled, a simplification noted among the limitations. For each request, we recorded patient age, sex, and department; clinical diagnosis; pre-transfusion hemoglobin (PRBC requests); coagulation parameters (FFP requests); platelet count and stated indication (PC requests); the number of units sought; and any documented symptoms supporting the request. Patient age and sex were recorded as routine request-level fields but were not analyzed or reported in this manuscript, as the audit's objectives centered on request appropriateness rather than demographic characterization; this is noted among the limitations. All identifying information was stripped before analysis began.

Appropriateness criteria

Each request was judged against component-specific benchmarks built from National Blood Transfusion Council (NBTC) India guidance, British Committee for Standards in Hematology (BCSH) recommendations, and the American Association of Blood Banks (AABB) Technical Manual; these criteria, together with the operational definitions applied, are summarized in Table 1 [2,4-6]. The PRBC threshold is additionally consistent with the restrictive strategy reaffirmed by the 2023 AABB International Guidelines for red blood cell transfusion [7]. These are prespecified operational audit criteria reflecting the guidance and local policy in institutional use during the study period, applied without modification from their published form, rather than a claim that they represent the single universally current threshold for every clinical context; more nuanced, procedure- and population-specific thresholds exist in some current guidance, and this is discussed further in the Limitations. Where a request could potentially be justified by more than one criterion, it was classified as appropriate if it satisfied any one of the listed conditions.

**Table 1:** Component-wise appropriateness criteria applied in the audit Symptomatic anemia was defined as exertional dyspnea, fatigue, tachycardia, or angina attributable to anemia; active bleeding as clinically evident hemorrhage requiring intervention; and invasive procedure as any surgical or bedside procedure carrying bleeding risk. These are prespecified operational audit criteria applied without modification from their published form (see Methods). Criteria adapted from NBTC India [2], BCSH [6], and the AABB Technical Manual [4]; the PRBC threshold is additionally consistent with the 2023 AABB International Guidelines for red blood cell transfusion [7]. Hb: hemoglobin; BP: blood pressure; PT: prothrombin time; aPTT: activated partial thromboplastin time; INR: international normalized ratio; PRBC: packed red blood cell; FFP: fresh frozen plasma; PC: platelet concentrate; NBTC: National Blood Transfusion Council; BCSH: British Committee for Standards in Hematology; AABB: American Association of Blood Banks

Component	Considered appropriate when	Marked inappropriate when
PRBC	Pre-transfusion Hb ≤8 g/dL or Hb >8 g/dL with documented hemodynamic compromise (heart rate >100/min or systolic BP <90 mmHg) or symptomatic anemia or anticipated significant surgical blood loss	Hb >8 g/dL without documented hemodynamic compromise, symptoms, or anticipated surgical loss
FFP	PT or aPTT >1.5× the upper limit of normal or INR >1.5, with active bleeding or before an invasive procedure, or activation of a massive transfusion protocol	Volume expansion or nutritional support; coagulopathy without bleeding or planned procedure; no coagulation workup documented
PC	Count <10,000/µL (prophylactic); <50,000/µL with active bleeding or before surgery; <100,000/µL before a neurosurgical or ophthalmic procedure	Count >50,000/µL in a non-bleeding, non-surgical patient; prophylaxis without bleeding or procedural risk

PRBC was considered justified if the pre-transfusion hemoglobin sat at or below 8 g/dL or exceeded that figure only where hemodynamic compromise was documented (heart rate above 100/min, systolic blood pressure under 90 mmHg, or symptomatic anemia) or where significant surgical blood loss was anticipated. This threshold follows NBTC India and AABB guidance [2,4].

FFP was considered justified where coagulopathy was objectively confirmed (prothrombin time (PT)/activated partial thromboplastin time (aPTT) beyond 1.5× the normal range or international normalized ratio (INR) over 1.5) in the presence of active bleeding or ahead of an invasive procedure or where a massive transfusion protocol (MTP) had been activated. Requests made purely to expand circulating volume or for nutritional reasons were marked as not meeting criteria, following BCSH recommendations [6]. Absence of a coagulation workup meant the required indication could not be confirmed, and such requests were classified as not meeting criteria on that basis; this reflects an absence of evidence for the criterion rather than an assumption of clinical wrongdoing, a distinction discussed further in the Results and Discussion.

Platelet (PC) was considered justified at a count below 10,000/µL given prophylactically, below 50,000/µL with active bleeding or ahead of surgery, or below 100,000/µL where a neurosurgical or ophthalmic procedure was planned, following standard AABB count-based criteria [4].

Two postgraduate pathology trainees scored every request independently. Where their judgments diverged, 147 instances in total (1.7%), a senior transfusion medicine specialist reviewed the case and arrived at a final consensus classification. The original per-reviewer classifications prior to adjudication were not retained in a form permitting calculation of a chance-corrected inter-rater agreement statistic (such as Cohen's kappa); only the overall disagreement rate is reported, and this is noted among the limitations.

Audit context

Data were analyzed as a single cross-sectional cohort spanning the full six-month window. During the study period, the department carried out its routine quality-improvement activities, including departmental feedback discussions, circulation of a component-specific transfusion-trigger reference card, and the addition of a mandatory free-text clinical indication field to the request form. Because these activities formed part of ongoing practice rather than a controlled experimental intervention, and because request-level dates were not retained in a form permitting reconstruction of their approximate timing within the six-month window, all requests were pooled for analysis, and no formal before-and-after or monthly comparison was undertaken. The possibility that these activities influenced prescribing during the window, in a manner this pooled analysis cannot resolve, is discussed further in the Limitations.

C:T ratio

The C:T ratio for PRBC requests was obtained by dividing the total units crossmatched by the total units actually transfused, calculated both for the study as a whole and separately by department. This is a unit-based measure, derived from crossmatch and transfusion unit records rather than from the request-level appropriateness dataset that forms the primary unit of analysis for this audit; the same distinction applies to the units-returned-unused figures. Department-level C:T ratios are reported only for Obstetrics and Gynecology, Surgery, and Oncology, because reliable, department-tagged crossmatch and transfusion unit counts were retained for these departments; equivalent department-level unit records were not consistently available for the remaining departments and are marked "NR" (not separately reported). A ratio at or below 2.0:1 is generally taken as the benchmark for efficient blood ordering; figures above this point reflect crossmatching in excess of genuine transfusion need, which strains inventory and increases the risk of units expiring unused.

Statistical analysis

Entries were logged in Microsoft Excel 2021 (Microsoft Corporation, Redmond, Washington, United States) and analyzed with IBM SPSS Statistics for Windows, Version 26.0 (IBM Corp., Armonk, New York, United States). Categorical data are reported as counts with percentages. The primary comparison was the overall association between department and appropriateness, tested with a single chi-squared test across all departments. Each department was then compared against the pooled remainder as a prespecified but exploratory secondary analysis, switching to Fisher's exact test where expected cell counts were small; these seven comparisons were adjusted for multiplicity using the Benjamini-Hochberg false discovery rate, with Bonferroni correction applied as a sensitivity check. These departmental comparisons are based on unadjusted, crude proportions; because appropriateness differs by component, departments with a different mix of PRBC, FFP, and platelet requests may appear more or less appropriate in part because of that mix rather than because of a true difference in prescribing quality, and no adjustment for component mix was performed. Proportions are accompanied by 95% CIs calculated using the Wilson method. Exact p-values are reported throughout; a value below 0.05 was taken as statistically significant.

The unit of analysis was the individual request, and a patient could contribute more than one request. Because the audit worked from de-identified, request-level records without a persistent patient identifier, requests could not be linked within patients, and clustered models such as generalized estimating equations or mixed-effects logistic regression could not be fitted. Requests were therefore analyzed as independent observations. This is a recognized limitation: where a subset of patients contributed several requests, the effective sample size is smaller than the request count implies, and the reported p-values, including the department-wise comparisons, should be regarded as anti-conservative (i.e., the true statistical significance is likely weaker than the nominal values suggest, and the precise magnitude of very small p-values should not be over-interpreted; the overall pattern of variation across departments is considered more robust than any individual p-value). This is revisited in the Limitations. No formal sample size calculation was performed: because every eligible request in the six-month window was analyzed (complete enumeration), the study is a census rather than a sample. With 8,752 requests, the overall appropriateness estimate of 70.9% carries a 95% CI of approximately 69.9-71.8% (±1.0%), indicating ample precision for the comparisons reported. Missing-data proportions for individual variables (diagnosis, laboratory values, units requested, and symptom documentation) were not separately tabulated as an independent completeness metric, since these fields were used directly to apply the appropriateness criteria in Table 1 rather than analyzed as standalone variables; this is noted among the limitations.

Ethics statement

This retrospective audit of existing, de-identified records was approved by the Sumandeep Vidyapeeth Institutional Ethics Committee on June 18, 2026 (approval number: SVIEC/UN/MEDI/RP/JUNE/26/69); individual consent was waived given the retrospective audit design and the use of fully de-identified data. 

## Results

Overview of blood component utilization

**Figure 2:**
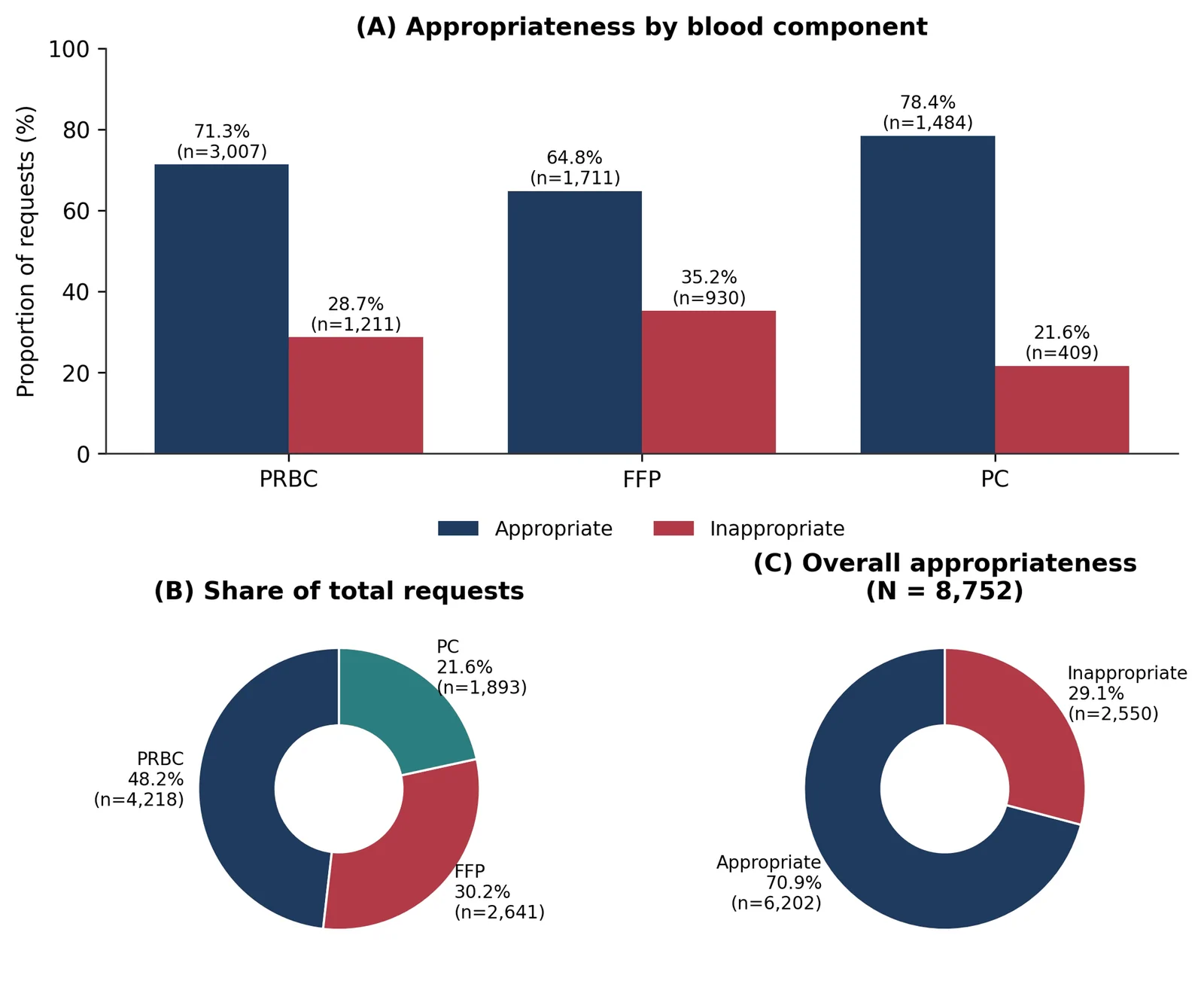
Blood component request utilization and appropriateness, with counts and percentages (A) Appropriate versus not-meeting-criteria rates among requests, by component, with FFP carrying the largest share not meeting criteria (35.2%). (B) Share of total requests contributed by each component. (C) Overall split among all 8,752 requests reviewed. PRBC: packed red blood cell; FFP: fresh frozen plasma; PC: platelet concentrate

Across the six-month window, 8,752 component requests were reviewed in total. PRBCs made up the largest share at 48.2% (n=4,218), with FFP contributing 30.2% (n=2,641) and PCs the remaining 21.6% (n=1,893). Taking all three components together, 70.9% of requests (6,202/8,752; 95% CI: 69.9-71.8) met the appropriateness criteria applied, leaving 29.1% (2,550) that did not. FFP requests had the lowest appropriateness, with 35.2% not meeting criteria, ahead of PRBC at 28.7% and PC at 21.6%. These figures, along with wastage and C:T data, are laid out in Table 2 and visualized in Figure 2.

**Table 2:** Summary of blood component utilization and request appropriateness Overall: 6,202/8,752 requests appropriate (70.9%; 95% CI: 69.9-71.8); 2,550 (29.1%) not meeting criteria. *Unit-based measures, derived from crossmatch and transfusion unit records rather than the request-level appropriateness dataset (see Methods) †Mean±SD Hb: hemoglobin; PRBC: packed red blood cell; FFP: fresh frozen plasma; PC: platelet concentrate; C:T: crossmatch-to-transfusion

Characteristic	PRBC	FFP	PC
Total requests reviewed, n	4,218	2,641	1,893
Units returned unused, n (%)*	312 (7.4%)	189 (7.2%)	97 (5.1%)
C:T ratio*	2.1:1	-	-
Mean pre-transfusion Hb (g/dL)†	6.8±1.4	-	-
Appropriate requests, n (%)	3,007 (71.3%)	1,711 (64.8%)	1,484 (78.4%)
Appropriate requests, 95% CI	69.9-72.6	62.9-66.6	76.5-80.2
Inappropriate requests, n (%)	1,211 (28.7%)	930 (35.2%)	409 (21.6%)

Departmental patterns of utilization

**Figure 3:**
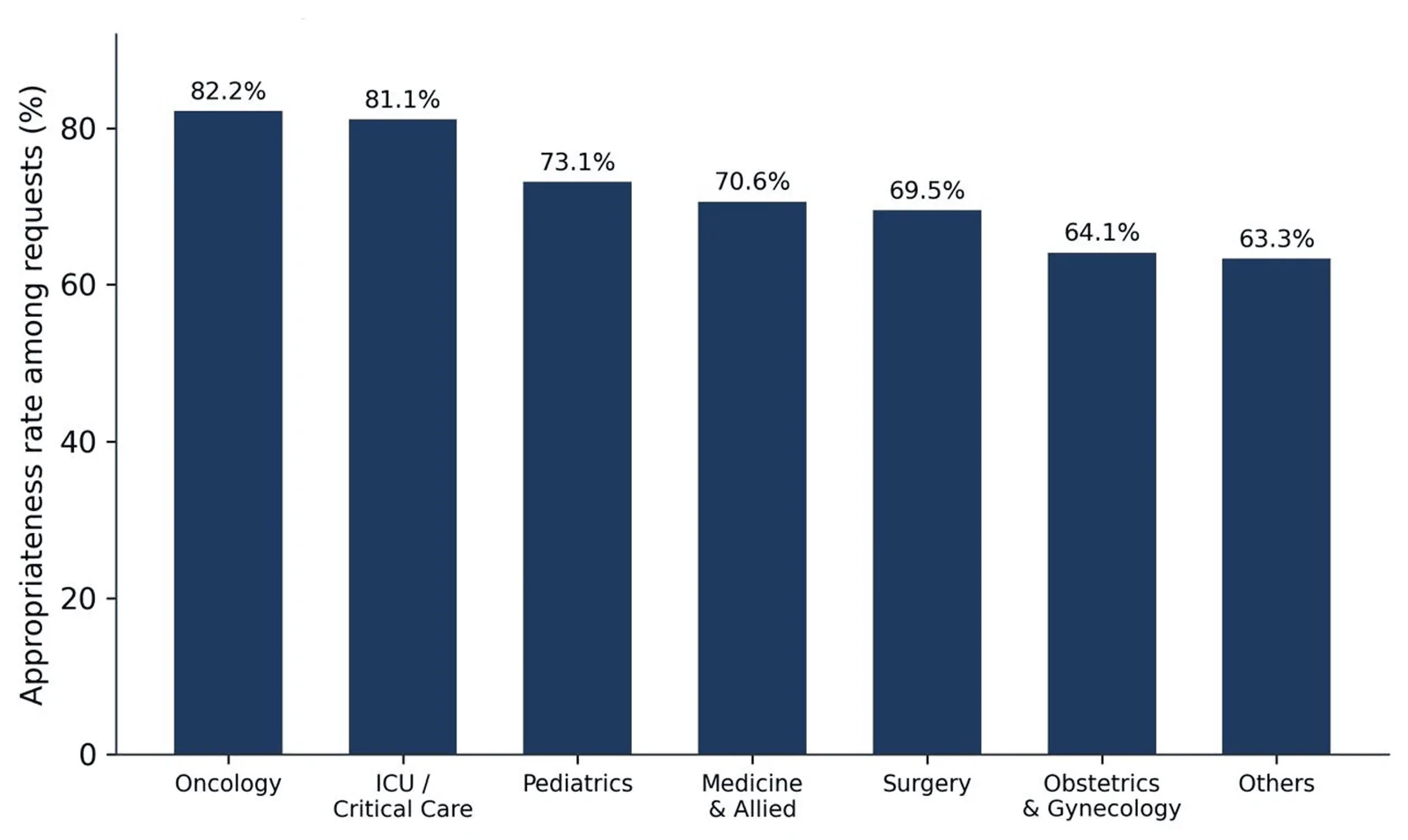
Department-wise appropriateness rates among blood component requests Oncology and ICU/Critical Care show the highest crude appropriateness; the "Others" group and Obstetrics and Gynecology the lowest. These are unadjusted for component mix and within-patient clustering and should not be read as a direct measure of prescribing quality (see Discussion and Limitations).

Appropriateness varied considerably by clinical area, and the overall association between department and appropriateness was statistically significant (χ²=161.3; df=6; p=3.2×10⁻³²), though this and the department-level p-values are unadjusted for within-patient clustering and are therefore best read as showing a clear, consistent gradient rather than as precise significance levels. These are also crude, unadjusted proportions that do not account for each department's mix of PRBC, FFP, and platelet requests, which itself differs by component-specific appropriateness; department rankings should therefore not be read as a direct measure of prescribing quality. Oncology (82.2%) and ICU/Critical Care (81.1%) sat at the top of the range; both differences from the pooled remainder remained significant after Benjamini-Hochberg correction. At the opposite end, the "Others" category, a mix of casualty and smaller specialty units, recorded the lowest appropriateness at 63.3%. Obstetrics and Gynecology, despite contributing the second-largest volume of requests, reached only 64.1% appropriateness. By contrast, the differences for Medicine, Surgery, and Pediatrics were not significant after correction. Full department-wise figures, with 95% CIs, PRBC C:T ratios, and false discovery rate-adjusted p-values, appear in Table 3 and Figure 3; possible explanations for this pattern are discussed, as hypotheses rather than demonstrated causes, in the Discussion.

**Table 3:** Department-wise blood component request volume and appropriateness These are crude, unadjusted proportions that do not account for each department's component mix and should not be read as a direct measure of prescribing quality (see Methods and Discussion). The overall association between department and appropriateness was the primary analysis (χ²=161.3; df=6; p=3.2×10⁻³²). *Department-level C:T ratios are shown where department-tagged red-cell ordering data were separately available; not available for the remaining departments. †The P column reports Benjamini-Hochberg FDR-adjusted values for the seven exploratory department-versus-remainder comparisons (chi-square with continuity correction); Bonferroni-adjusted values were consistent, with every significant comparison remaining significant. These p-values are not adjusted for possible within-patient clustering and may be anti-conservative; exact magnitudes should not be over-interpreted (see Statistical Analysis and Limitations). C:T: crossmatch-to-transfusion ratio (packed red cells); NR: not separately reported; FDR: false discovery rate

Department	Total requests	Appropriate n (%)	Inappropriate n (%)	95% CI	PRBC C:T*	P (FDR)†
Medicine and Allied	2,314	1,634 (70.6%)	680 (29.4%)	68.7-72.4	NR	0.78
Obstetrics and Gynecology	1,847	1,184 (64.1%)	663 (35.9%)	61.9-66.3	2.6:1	2.6×10⁻¹²
Surgery	1,563	1,086 (69.5%)	477 (30.5%)	67.2-71.7	2.4:1	0.23
Oncology	1,102	906 (82.2%)	196 (17.8%)	79.8-84.4	1.6:1	7.1×10⁻¹⁸
Pediatrics	687	502 (73.1%)	185 (26.9%)	69.6-76.3	NR	0.23
ICU/Critical Care	594	482 (81.1%)	112 (18.9%)	77.8-84.1	NR	3.4×10⁻⁸
Others	645	408 (63.3%)	237 (36.7%)	59.5-66.9	NR	2.1×10⁻⁵
Total	8,752	6,202 (70.9%)	2,550 (29.1%)	69.9-71.8	2.1:1	-

Reasons for requests not meeting appropriateness criteria

**Figure 4:**
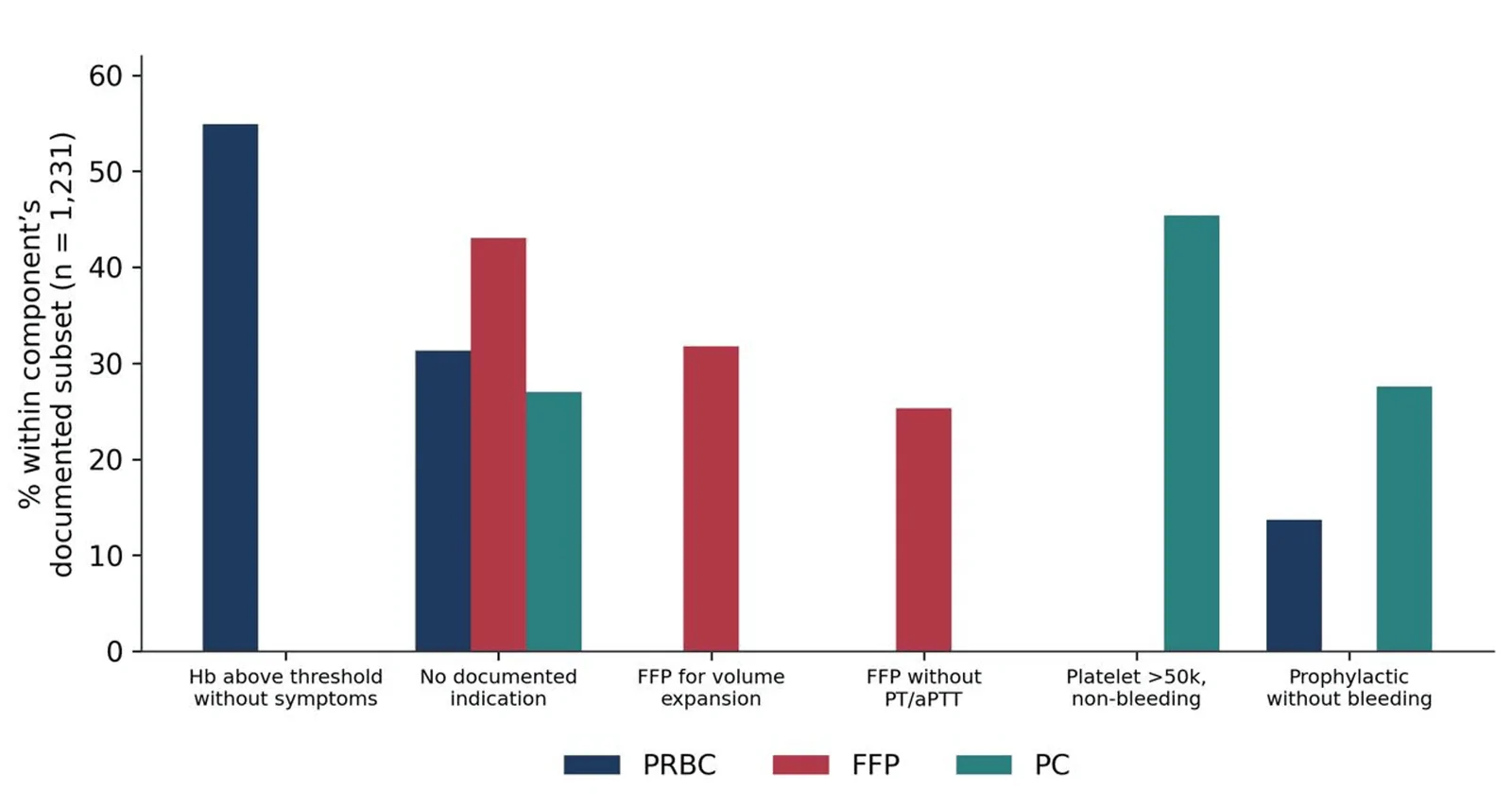
Documented primary reason among blood component requests not meeting appropriateness criteria, by component Bars show the percentage within each component's documented subset and apply only to the 1,231 requests with a single documented reason (568 PRBC, 467 FFP, 196 PC), not to all requests not meeting criteria. PRBC: packed red blood cell; FFP: fresh frozen plasma; PC: platelet concentrate; Hb: hemoglobin; PT: prothrombin time; aPTT: activated partial thromboplastin time

A single primary reason was documented for 1,231 of the 2,550 requests that did not meet appropriateness criteria (48.3%), and these are summarized in Table 4 and Figure 4. Within this documented subset, the biggest driver for PRBC was transfusing at a hemoglobin level above the agreed cut-off without any accompanying hemodynamic symptoms, accounting for 54.9% of documented PRBC reasons. For FFP, the picture was different: 31.7% of documented reasons were requests made purely to expand volume with no coagulopathy on record, and a further 25.3% lacked the mandatory PT/aPTT workup altogether, the latter reflecting the absence of an evidence base for the criterion, as described in the Methods, rather than a documented clinical error. For PCs, the dominant issue was a count above 50,000/µL in patients who were neither bleeding nor headed for surgery, responsible for 45.4% of documented platelet reasons.

**Table 4:** Documented primary reason among blood component requests not meeting appropriateness criteria A single primary reason was assigned from the request form and clinical record where one was clearly documented and distinct from a general absence of a stated indication (see "No documented clinical indication on request form" above); the remaining requests not meeting criteria either carried more than one contributing reason or lacked a clearly recorded primary reason. Because a retrospective absence of documentation does not on its own establish the absence of a valid clinical indication, this latter group is labelled indeterminate rather than confirmed inappropriate and is shown as a separate row so that the column totals reconcile exactly with the counts not meeting criteria in Tables 2-3 (1,211 PRBC, 930 FFP, 409 PC). Percentages are calculated within each component's documented subset (568 PRBC, 467 FFP, 196 PC; 1,231 in total). Hb: hemoglobin; PT: prothrombin time; aPTT: activated partial thromboplastin time

Reason	PRBC n (%)	FFP n (%)	PC n (%)
Hb above threshold (>8 g/dL) without hemodynamic symptoms	312 (54.9%)	-	-
No documented clinical indication on request form	178 (31.3%)	201 (43%)	53 (27%)
FFP for volume expansion without documented coagulopathy	-	148 (31.7%)	-
FFP without pre-transfusion PT/aPTT documentation	-	118 (25.3%)	-
Platelet count >50,000/µL in non-surgical, non-bleeding patient	-	-	89 (45.4%)
Prophylactic transfusion without active or anticipated bleeding	78 (13.7%)	-	54 (27.6%)
Subtotal with a documented primary reason, n	568	467	196
Indeterminate: no single primary reason documented, n	643	463	213
Total not meeting criteria, n	1,211	930	409

Two related but distinct categories should not be conflated. "No documented clinical indication on request form" (Table 4) refers to a specific documented reason applied when the request itself carried no stated clinical indication. "No single primary reason documented" (Table 4, 1,319 requests, 51.7%) is a separate, larger category comprising requests that either carried more than one contributing reason or for which no primary reason could be clearly assigned at all. Because a retrospective absence of documentation does not, on its own, prove the absence of a valid clinical indication, we consider this latter group indeterminate due to insufficient documentation rather than confirmed instances of inappropriate clinical decision-making; a structured breakdown of these requests would require a level of free-text capture that the current forms did not consistently provide. The predominance of missing or incomplete documentation in this group is itself a substantive finding, pointing to a documentation gap that runs alongside, and is not always separable from, a prescribing gap.

C:T ratio

Taking the study period as a whole, the PRBC C:T ratio worked out to 2.1:1. At the department level, Obstetrics and Gynecology (2.6:1) and Surgery (2.4:1) were the heaviest contributors to unnecessary crossmatching, while Oncology posted the most efficient figure at 1.6:1, unsurprising given the relatively predictable transfusion needs of patients with chronic, treatment-related anemia. As noted in the Methods, department-level C:T data were available for only these three departments.

## Discussion

Roughly three in 10 blood component requests in this six-month audit fell short of established clinical criteria, with FFP carrying the largest share of that shortfall (35.2% not meeting criteria). Reported appropriateness varies widely with setting and criteria: a South Indian audit found red cell appropriateness as high as 90% [8], whereas an international consensus exercise judged only about 40% of allogeneic red cell transfusions clearly appropriate [9]. Component-usage patterns themselves differ substantially between Indian centers [10], and reviews of transfusion practice underscore how often use departs from accepted indications [11]. Inappropriate FFP use has likewise been substantial elsewhere: around half of FFP transfusions were inappropriate at a North Indian trauma center [12], and a large audit in England found 43% of FFP given without documented bleeding [13], with FFP repeatedly identified as the component most prone to inappropriate use. Our own overall appropriateness figure of 70.9% sits within this reported spread, but nearly a third of requests not meeting criteria is not a result that institutions can be comfortable with. The contribution here lies less in that headline rate, which is consistent with prior reports, than in the breadth and granularity behind it: a hospital-wide view spanning all three major components that localizes requests not meeting criteria to specific departments and, where documented, specific reasons.

The scale of FFP requests not meeting criteria is worth dwelling on. FFP is sometimes used without the same threshold-based scrutiny applied to red cells or platelets, despite carrying its own transfusion risks, among them transfusion-related acute lung injury (TRALI), transfusion-associated circulatory overload (TACO), and alloimmunization [14]. Transfusion guidelines have long been explicit that FFP should not be used for volume support or for mild coagulopathy in the absence of bleeding [6], yet our data show this advice is still routinely set aside. More than a quarter of FFP requests carried no PT/aPTT result at all; as discussed above, this was treated as an absence of evidence for the required indication rather than direct proof of inappropriate practice, but the pattern nonetheless points to a documentation gap running alongside, and difficult to separate from, a clinical judgment gap, a pattern also noted in earlier Indian blood-usage audits [10].

The spread of appropriateness scores across departments tells its own story, though the explanation for it remains a hypothesis rather than a demonstrated finding of this audit. One plausible interpretation, consistent with the observed pattern but not directly tested here, is that ICU and oncology, both areas with more standardized internal protocols and closer day-to-day oversight in many institutions [7], benefit from more consistent prescribing behavior, while casualty and the mixed "Others" group, where requests are often raised under greater time pressure, by relatively junior staff, and without easy access to a transfusion specialist for a second opinion, do not. We did not measure staffing seniority, time pressure, or access to specialist advice, and, as noted in the Methods and Results, these departmental comparisons are also unadjusted for each department's component mix. Any explanation involving these factors should therefore be treated as hypothesis-generating for this institution rather than as an established, generalizable cause. If confirmed by further work, closing this gap would likely require point-of-care decision support and a clearer route to on-call transfusion medicine advice in exactly these settings.

An overall C:T ratio of 2.1:1 still sits a little above the commonly cited optimal mark of 2.0:1 or below [15]. Crossmatching well beyond actual transfusion need is not a neutral inefficiency; it locks up inventory and raises the odds that reserved units will simply expire unused. Adopting maximum surgical blood ordering schedules (MSBOS) and type-and-screen protocols for lower-risk elective cases, as recommended by both the AABB and the National Institute for Health and Care Excellence (NICE) blood transfusion guideline, would likely tighten this figure further [16].

Taken together, these findings make a case worth considering for embedding structured audit and feedback as a standing process rather than a one-off exercise. Non-punitive, transparent feedback of this kind has repeatedly been shown to shift prescribing behavior across healthcare settings, including in a Cochrane systematic review of audit-and-feedback interventions [17,18], and is well suited to the gaps identified here, though its specific effect at this institution was not tested by the present study. The absence of an electronic ordering system with built-in transfusion prompts means adherence at present rests on individual memory rather than any system-level nudge; where such computerized physician order entry (CPOE) systems with decision support have been introduced elsewhere, inappropriate transfusion rates have fallen substantially [19]. There is also a more structural limitation to criteria built around a single number: patients whose hemoglobin sits just above the cut-off but who are genuinely symptomatic may be wrongly flagged as not meeting criteria, pointing to value in pairing future audits with a validated symptom score rather than hemoglobin alone and in testing more nuanced, procedure- and population-specific thresholds such as those in current AABB and International Collaboration for Transfusion Medicine Guidelines (ICTMG) guidance, which this audit's uniform ≤8 g/dL PRBC threshold did not incorporate; a sensitivity analysis re-classifying PRBC requests against a stricter, general-population 7 g/dL threshold was not possible with the aggregated data available for this audit and is recommended for future work with patient-level data retention. The case for tighter stewardship is reinforced by recent experience with supply-side shocks to the blood system, which have shown how quickly demand-side waste becomes unaffordable once donation volumes are disrupted [20].

What this study adds is breadth and resolution. Where most Indian appropriateness audits are retrospective and confined to a single component, a single department, or a few hundred transfusions, this audit examined all three major components across every clinical department in 8,752 requests and identified both where practice diverges between departments and the specific, documented reasons requests fail against criteria. These findings should be regarded as hypothesis-generating for this institution rather than as causally established or directly generalizable conclusions, given the limitations below.

Several limitations concern the study's statistical and methodological design. Requests could not be linked within patients, preventing modelling of within-patient clustering. Analysis of independent requests likely overstates the precision of reported p-values; therefore, very small p-values should not be over-interpreted. The overall pattern of departmental variation is considered more robust than the exact statistical significance of any individual comparison. The two initial assessors' individual classifications prior to adjudication were not retained, so a chance-corrected inter-rater agreement statistic, such as Cohen's kappa, could not be calculated; only the raw disagreement rate (147/8,752, 1.7%) is reported. Departmental comparisons are based on unadjusted crude proportions that do not account for each department's component mix and should not be read as a direct reflection of prescribing quality. The uniform ≤8 g/dL PRBC threshold applied throughout is more liberal than the general-population 7 g/dL threshold recommended by current AABB guidance for hemodynamically stable adults without a qualifying risk factor; a sensitivity analysis using this stricter threshold was not possible with the data retained for this audit. No formal sample size calculation was performed, as this was a complete-enumeration census rather than a sampled study.

Several further limitations concern the underlying data. The study was conducted at one center and relied on what was written down, so undocumented clinical reasoning is not captured. A single documented primary reason could be assigned for only 1,231 of the 2,550 requests not meeting criteria, and the remaining 1,319 are best regarded as indeterminate due to insufficient documentation rather than confirmed inappropriate use, so the reason-level breakdown in Table 4 characterizes roughly half of these requests rather than all of them. Missing-data proportions for individual variables such as diagnosis, laboratory values, and symptom documentation were not separately tabulated as an independent completeness metric. Patient age and sex were recorded at extraction but were not analyzed or reported, as demographic characterization fell outside this audit's scope. Department-level C:T ratios and unit-returned data were available for only three of seven departments, owing to inconsistent department-tagged unit records. Routine quality-improvement activity during the study window may have influenced prescribing. Because request-level dates were not retained, the data were analyzed as a single cross-section, and any temporal shift within the window cannot be resolved; this pooling should be borne in mind when interpreting the overall appropriateness estimate as representative of any single point in time. Finally, the audit stopped short of tracking patient-level clinical outcomes, transfusion reactions, length of stay, and infection rates, that would have linked appropriateness more directly to clinical benefit.

## Conclusions

This audit found that a sizeable share of blood component requests, FFP requests in particular, did not meet the prespecified evidence-based criteria applied at this institution and that appropriateness varied markedly across clinical departments and components. As discussed above, these findings are best regarded as descriptive and hypothesis-generating for this institution: the retrospective, documentation-dependent, single-center design and the unadjusted departmental comparisons mean the reasons behind this pattern, whether gaps in prescriber knowledge, documentation practice, or institutional systems, cannot be firmly established from this study alone and should be treated as plausible interpretations rather than demonstrated causes.

Building on these findings, we would propose the consideration of a permanent hospital transfusion committee with a standing brief to run component-specific audits, track the C:T ratio regularly, deliver recurring transfusion training to junior medical staff, and work toward digital transfusion triggers within the hospital's existing information systems. We emphasize that these are policy recommendations informed by the findings of this audit, not interventions whose effectiveness was tested here. A natural next step would be multicenter, prospective work that retains patient-level data, enabling clustering-adjusted analysis, component mix-adjusted departmental comparisons, and threshold sensitivity analyses, and that follows patients beyond the request decision itself, capturing reactions, length of stay, and infection outcomes, to more firmly establish what appropriateness-focused audit and feedback actually buys in terms of patient safety within the Indian tertiary care setting.
